# Through‐Space Conjugated Electron Transport Materials for Improving Efficiency and Lifetime of Organic Light‐Emitting Diodes

**DOI:** 10.1002/advs.202200374

**Published:** 2022-03-24

**Authors:** Pingchuan Shen, Hao Liu, Zeyan Zhuang, Jiajie Zeng, Zujin Zhao, Ben Zhong Tang

**Affiliations:** ^1^ State Key Laboratory of Luminescent Materials and Devices Guangdong Provincial Key Laboratory of Luminescence from Molecular Aggregates South China University of Technology Guangzhou 510640 China; ^2^ Shenzhen Institute of Aggregate Science and Technology School of Science and Engineering The Chinese University of Hong Kong Shenzhen Guangdong 518172 China; ^3^ AIE Institute Guangzhou Development District Huangpu Guangzhou 510530 China

**Keywords:** electron transport, multi‐dimensional transport, operational lifetime, organic light‐emitting diode, through‐space conjugation

## Abstract

Thermally stable electron transport (ET) materials with high electron mobility and high triplet state energy level are highly desired for the fabrication of efficient and stable organic light‐emitting diodes (OLEDs). Herein, a new design strategy of constructing through‐space conjugated folded configuration is proposed to explore robust ET materials, opposite to the widely used planar configuration. By bonding two quinolines to the 9,10‐positions of phenanthrene, two novel folded molecules with high thermal and morphological stabilities and high triplet state energy levels (>2.7 eV) are created. These folded molecules possess excellent ET ability with electron mobilities of three orders of magnitude higher than those of linear and planar counterparts. Theoretical calculation and crystallography analysis demonstrate the through‐space conjugated folded configuration has not only reduced reorganization energy but also enlarged charge transfer integral at various dimensions, bringing about efficient multi‐dimensional ET, independent of molecular orientation. By adopting the folded molecule as ET layers, OLEDs with no matter delayed fluorescence or phosphorescence emitters can achieve high external quantum efficiencies and long operational lifetimes simultaneously. This work paves a new avenue towards robust ET materials to improve efficiency and stability of OLEDs.

## Graphical abstract

Multi‐dimensional electron transport materials with a through‐space conjugated folded configuration are explored, exhibiting higher triplet energy levels and higher electron mobilities than through‐bond conjugated linear counterparts. The efficiency and lifetime of organic light‐emitting diodes are improved by using them as electron transport layers.

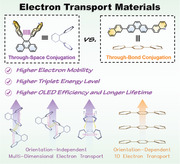

## 1 Introduction

Organic light‐emitting diode (OLED) has achieved impressive progress since its innovation in 1987, which is regarded as a highly promising technique for next‐generation display and illumination.^[^ ^1^ ^]^ Balancing carrier transport is of significant importance to improve electroluminescence (EL) efficiency and operational lifetime of OLED.^[^ ^2^ ^]^ However, due to electron‐rich nature, purely organic materials often have better hole transport than electron transport (ET). Besides, an ideal ET molecule is required to have high triplet state energy level (*E*_T_) to confine electrogenerated excitons within emitting layer. Introducing rigid polycyclic electron‐deficient moieties and building a planar *π*‐conjugated system are widely adopted in design of ET molecule.^[^ ^3^ ^]^ But, generally, the *E*_T_ of the resulting molecule is relatively low because of the well *π*‐conjugated molecular backbone.^[^ ^4^ ^]^ Decreasing conjugation of molecular backbone is helpful to increase *E*_T_,^[^ ^5^ ^]^ but meanwhile, it may also reduce molecular rigidity, resulting in lowered glass‐transition temperatures, which is detrimental to device lifetime.^[^ ^2b^ ^]^ Moreover, most developed ET molecules have linear or planar structures and their ET abilities are highly dependent on molecular orientation.^[^ ^6^ ^]^ However, molecular orientation control is quite troublesome with very limited techniques.^[^ ^7^ ^]^ Due to these contradictions, it still remains challenging to achieve good thermal stability, high *E*_T_, and orientation‐independent fast ET at the same time for a conventional well‐conjugated molecule.^[^ ^8^ ^]^

**Figure 1:**
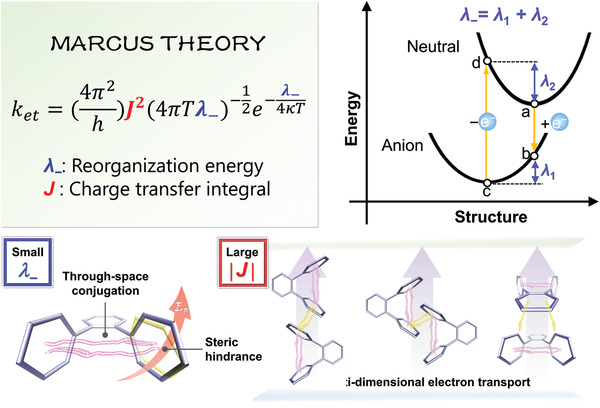
Schematic illustrations of the strategy of developing through‐space conjugated ET molecules. Upper‐right plot is the illustration of the four‐points scheme for the reorganization energy calculation. *E*_p_ in bottom‐left plot stands for the intramolecular rotation potential of aryl groups in ET molecules.

On the basis of Marcus electron transfer theory, intermolecular electron transfer rate (*k*_et_) can be estimated according to the equation shown in **Figure** **1**, in which *h* is Plank constant, *k* is Boltzmann constant, *T* is temperature, *λ*_−_ is reorganization energy, and *J* is charge transfer integral between two molecules. At a given *T*, the *k*_et_ is determined by *λ*_−_ and *J* ^2^. Obviously, a small *λ*_−_ and a large absolute value of *J*, namely |*J*|, benefit electron transfer. Since *λ*_−_ is the sum of *λ*_1_ and *λ*_2_, in which *λ*_1_ and *λ*_2_ are the stabilization energy in geometry relaxation of anion radical and neutral ET molecules, respectively, the harder geometrical change during the process of accepting and losing an electron, the smaller *λ*_−_ and thus larger *k*_et_ can be achieved, that is, molecular structural rigidification is conducive to ET. Taking these factors into consideration, in this work, as a proof of concept, we propose a new design strategy for ET molecules of constructing a through‐space conjugated folded structure to increase molecular rigidity and thus *λ*_−,_ and to shorten through‐bond conjugation to acquire high *E*_T_. Besides, the combination of through‐space conjugation and through‐bond conjugation facilitates effective multi‐dimensional intermolecular interaction, leading to large |*J*| values between adjacent molecules. As a result, the through‐space conjugated ET molecules have large electron mobilities (*μ*_e_s), independent of molecular orientation (Figure 1).^[^ ^9^ ^]^

**Figure 2:**
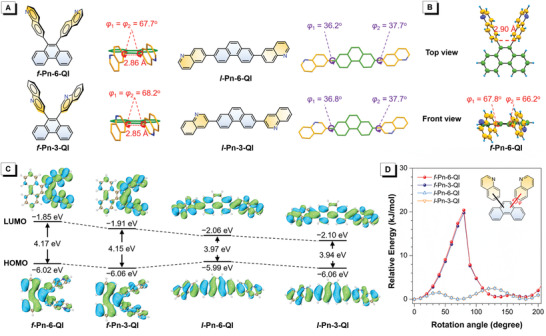
A) Chemical structures and theoretically optimized geometries (ground state) of *f*‐Pn‐6‐Ql, *f*‐Pn‐3‐Ql, *l*‐Pn‐6‐Ql, and *l*‐Pn‐3‐Ql. B) Crystal structures of *f*‐Pn‐6‐Ql. C) Frontier molecular orbitals contours and energy levels (ground state) of *f*‐Pn‐6‐Ql, *f*‐Pn‐3‐Ql, *l*‐Pn‐6‐Ql, and *l*‐Pn‐3‐Ql. D) Conformational energy profile for the rotation of quinoline groups of *f*‐Pn‐6‐Ql, *f*‐Pn‐3‐Ql, *l*‐Pn‐6‐Ql, and *l*‐Pn‐3‐Ql.

The tailor‐made folded molecules (*f*‐Pn‐6‐Ql and *f*‐Pn‐3‐Ql) are created by connecting two electron‐deficient quinolines to the 9,10‐positions of polycyclic planar phenanthrene that has intrinsic high *E*_T_ of over 2.7 eV (**Figure** **2**A).^[^ ^10^ ^]^ Delightfully, it is found that the *μ*_e_s of both through‐space conjugated folded molecules are about three orders of magnitudes higher than their linear counterparts (*l*‐Pn‐6‐Ql and *l*‐Pn‐3‐Ql). What is more, the OLEDs fabricated with *f*‐Pn‐6‐Ql as ET layers can attain not only better EL efficiencies but also longer operational lifetime than the device using *l*‐Pn‐6‐Ql as ET layer. No matter using delayed fluorescence or phosphorescence emitters, the OLEDs using *f*‐Pn‐6‐Ql as ET layer can also provide high EL efficiencies and long operational lifetimes simultaneously, which is quite difficult for the OLEDs using commercialized ET materials like 1,3,5‐tri[(*m*‐pyrid)‐3‐yl‐phenyl)]‐benzene (TmPyPB) and 4,7‐diphenyl‐1,10‐phenanthroline (BPhen). The proposed ET molecule design is of high importance for the development of robust carrier transport materials for high‐performance OLEDs.

## 2 Result and Discussion

The delicately folded molecules and their linear counterparts are synthesized in one step via Suzuki–Miyaura coupling (Scheme S1, Supporting Information). *f*‐Pn‐6‐Ql can be obtained facilely in a high yield of 89.8%, while its isomer *f*‐Pn‐3‐Ql only has a low yield of 5.1%. It is believed that the substitution reactions occurring at the 9‐ and 10‐positions of 9,10‐dibromophenanthrene are not simultaneous. When 9,10‐dibromophenanthrene is substituted by one 3‐quinoline at the 9‐position, the *N* atom of quinoline is very close to the reaction site of the 10‐position of phenanthrene. This *N* atom may interact with palladium catalyst and thus interfere with the subsequent oxidative addition or reductive elimination processes of palladium catalyst at the 10‐position of phenanthrene, leading to a poor yield of *f*‐Pn‐3‐Ql. The purity of folded molecules is carefully examined by NMR, high‐resolution mass spectra, and high‐performance liquid chromatography spectra (Figure S2, Supporting Information). The detailed synthetic procedures and characterization data are provided in Supporting information. Both *f*‐Pn‐6‐Ql and *f*‐Pn‐3‐Ql have good thermal stability with high decomposition temperatures of 342 and 360 °C (Figure S3A, Supporting Information), respectively. No glass‐transition temperatures are detected by differential scanning calorimetry (Figure S3B, Supporting Information), indicative of good morphological stability. In comparison with linear counterparts, the folded molecules are found to have larger optical gaps and higher *E*_T_s (**Table** **1**). According to the phosphorescence spectra in neat films (Figures S4 and S5, Supporting Information), the *E*_T_s of *f*‐Pn‐6‐Ql and *f*‐Pn‐3‐Ql are calculated to be 2.72 and 2.69 eV, respectively, which are close to that of TmPyPB (2.75 eV)^[^ ^5a^ ^]^ and higher than those of *l*‐Pn‐6‐Ql (2.52 eV), *l*‐Pn‐3‐Ql (2.53 eV) and BPhen (2.60 eV).^[^ ^3a^ ^]^ The *E*_T_s of both folded molecules are comparable or even higher than those of most reported ET materials with fused polycyclic arenes as skeletons (<2.7 eV)^[^ ^3^, ^13^ ^]^ enable them to better confine electrogenerated excitons within emitting layer. The lowest unoccupied molecular orbitals (LUMOs) play a key role in ET, and thus the energy levels of LUMOs can exert great impacts on ET ability. Cyclic voltammograms (Figure S3C, Supporting Information) show that *f*‐Pn‐6‐Ql and *f*‐Pn‐3‐Ql have low‐lying LUMO energy levels of −2.41 and −2.56 eV, respectively, which ensure low electron‐injection barrier for them. All of these experiment data indicate *f*‐Pn‐6‐Ql and *f*‐Pn‐3‐Ql are promising to serve as ET materials.

**Table 1:** Energy levels and electron mobility of *f*‐Pn‐6‐Ql, *f*‐Pn‐3‐Ql, *l*‐Pn‐6‐Ql, and *l*‐Pn‐3‐Ql

	Experimental data						Theoretical data				
	*E*_HOMO_ ^a)^ [eV]	*E*_LUMO_ ^a)^ [eV]	*E*_g_ ^b)^ [eV]	*E*_S_ ^c)^ [eV]	*E*_T_ ^c)^ [eV]	*μ_e_* ^d)^ [cm^2^ V^−1^ s^−1^]	*E*_HOMO_ ^e)^ [eV]	*E*_LUMO_ ^e)^ [eV]	*λ*_1_ ^f)^ [eV]	*λ*_2_ ^f)^ [eV]	*λ*_−_ ^f)^ [eV]
*f*‐Pn‐6‐Ql	−5.91	−2.41	3.58	3.54	2.72	2.6 × 10^−3^	−6.02	−1.85	0.082	0.154	0.235
*f*‐Pn‐3‐Ql	−5.98	−2.56	3.58	3.64	2.69	2.3 × 10^−5^	−6.06	−1.91	0.062	0.105	0.167
*l*‐Pn‐6‐Ql	−5.76	−2.55	3.41	3.44	2.52	1.8 × 10^−6^	−5.99	−2.05	0.110	0.175	0.285
*l*‐Pn‐3‐Ql	−5.85	−2.59	3.36	3.26	2.53	5.4 × 10^−9^	−6.06	−2.10	0.118	0.175	0.293

^a)^ HOMO and LUMO energy levels estimated by the onsets of oxidation and reduction peaks in cyclic voltammograms

^b)^ Optical bandgaps calculated from the absorption edges of the UV–vis absorption spectra

^c)^ Lowest singlet excited state energy level (*E*_S_) and lowest triplet state energy level (*E*_T_) obtained from the onsets of fluorescence and phosphorescence spectra in neat films, respectively

^d)^ Electron mobility at an electric field of 6.4 × 10^5^ V cm^−1^

^e)^ HOMO and LUMO energy levels calculated by DFT methods (B3LYP‐D3(BJ)/def2‐TZVP)^[^ ^11^ ^]^ based on the optimized geometries in ground state

^f)^ *λ*_1_ is stabilization energy by the geometry relaxation of anion radical; *λ*_2_ is stabilization energy by the geometry relaxation of neutral molecule; Reorganization energy *λ*_−_ = *λ*_1_ + *λ*_2_. Related single point calculations based on DFT methods are carried out at M06‐2X/ma‐QZVP level.^[^ ^12^ ^]^

The crystallography analysis reveals that *f*‐Pn‐6‐Ql holds a highly twisted conformation with large dihedral angles of 66.2^o^ and 67.8^o^ between quinoline and phenanthrene planes (Figure 2B) in crystals. The two quinoline groups are aligned face to face with a shortest inter‐plane distance of 2.90 Å, which is defined by the shortest C—C distance between two aryl stacked groups. And strong *π*–*π* interactions with short inter‐plane distances of 3.36–3.47 Å are also found between quinoline groups of adjacent molecules. These distances are shorter than the sum of van der Waals radii of two sp^2^ C atoms (3.5 Å), implying the presence of multiple long‐term strong electronic coupling among quinoline groups. In addition, abundant intermolecular C—H∙∙∙*π* interactions with short distances of 2.39–2.65 Å are also observed between quinoline and phenanthrene groups of adjacent molecules. All of these intermolecular interactions can not only rigidify molecular structures but also provide opportunity for efficient multi‐dimensional ET.

To investigate the configuration difference between folded and linear molecules, theoretical calculation using density functional theory (DFT) with the method of B3LYP‐D3(BJ)/def2‐TZVP are carried out to obtain their optimized geometries at ground state. As shown in Figure 2A, for folded molecules, the torsion angles between quinoline and phenanthrene are as large as ≈68^o^, which are much larger than those of linear counterparts (≈37^o^). Two quinoline groups are stacked in a face‐to‐face manner with the shortest C—C distance of ≈2.85 Å, and apparent orbitals overlap can be found in LUMOs (Figure 2C), demonstrating strong through‐space conjugation between quinoline groups.^[^ ^14^ ^]^ To quantitatively evaluate the rigidity of folded and linear molecules which holds significantly impacts on *λ*_−_, relaxed potential energy surface scan is carried out. Concerning the high rigidity of phenanthrene and quinoline groups, the geometry relaxation of these molecules should be dominated by the rotation of quinoline groups. As shown in Figure 2D, the energy barriers for rotating quinoline groups are calculated to be about 20 kJ mol^−1^ for folded molecules, 10‐fold higher than those of linear counterparts (2 kJ mol^−1^), that is, it is much more difficult for folded molecules to change geometries during ET process. The *λ*_−_ values of *f*‐Pn‐3‐Ql and *f*‐Pn‐6‐Ql are calculated to be 0.167 and 0.235 eV, respectively, smaller than those of *l*‐Pn‐3‐Ql and *l*‐Pn‐6‐Ql (0.293 and 0.285 eV).

**Figure 3:**
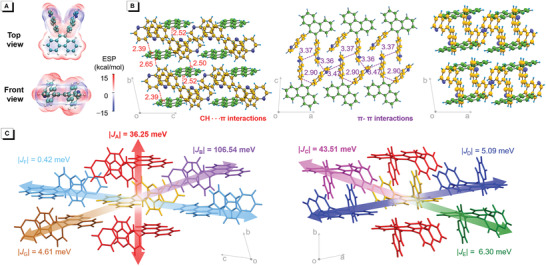
A) Isosurface of ESP for *f*‐Pn‐6‐Ql. B) Packing arrangements of *f*‐Pn‐6‐Ql in crystal. C) Calculated |*J*| values for *f*‐Pn‐6‐Ql. |*J*| values at different directions are labeled in different colors.

On the other hand, |*J*| is greatly affected by the intermolecular distance and orientation. Electrostatic potential (ESP) plays an essential role in determining intermolecular interactions, which is widely used to understand or predict the intermolecular packing modes in aggregate states.^[^ ^15^ ^]^ Due to large intramolecular steric hindrance in folded molecules, the electronegative regions on phenanthrene and quinoline groups face two nearly orthogonal directions, while the electropositive regions are distributed in almost all directions around the molecules, suggesting folded molecules could have effective multi‐dimensional intermolecular interactions (**Figure** **3**A). In comparison with folded molecules, the ESP distribution on slab‐like linear molecule is more concentrated. As shown in Figure S6A, Supporting Information, taking *l*‐Pn‐6‐Ql as a linear slab, the electonegative region is distributed on the upper and lower surface of the slab, while the electropositive region is distributed on the edge of the slab. Such a concentrated ESP distribution tends to make molecules align at specific direction, leading to the anisotropy of ET.

To prove the multi‐dimensional ET feature of folded molecules, the dimers in the crystals of *f*‐Pn‐6‐Ql are selected to calculate the |*J*| values based on the intermolecular couplings of LUMOs.^[^ ^16^ ^]^ Concerning the thermal factors that have significant effect on ET processes, thermal fluctuation energy *kT* (*kT* ≈ 25 meV at room temperature; *k* is Boltzmann constant; *T* is temperature) can be roughly taken as an energy barrier in ET processes. At a given temperature *T*, |*J*| ≫ *kT* means ET can easily occur; |*J*| ≈ *kT* means ET can be readily affected by thermal fluctuation; |*J*| ≪ *kT* means ET is very ineffective.^[^ ^17^ ^]^ As shown in Figure S7, Supporting Information, in the crystal of *f*‐Pn‐6‐Ql, for a chosen central molecule, seven different dimers can be found according to the relative intermolecular positions between central molecules and 10 nearest periphery molecules. The |*J*| values in dimers A, B, and C that are located in three different directions of the central molecules are calculated to be 36.25, 106.54, and 43.51 meV, respectively, much larger than *kT* value at room temperature, demonstrating that these dimers can be effective ET channels (Figure 3C). Strong intermolecular *π*–*π* or C—H∙∙∙*π* interactions in these dimers should be the main cause for their large |*J*| values. To further illustrate the multi‐dimensional ET properties of folded molecules, single crystals of linear molecule *l*‐Pn‐6‐Ql are also obtained to serve as a control. There are five different dimers in the crystals of *l*‐Pn‐6‐Ql (Figure S8, Supporting Information) and the dimer *β* has the largest |*J*| value of 61.86 meV (Figure S6B, Supporting Information), which is still much smaller than that in dimer B of *f*‐Pn‐6‐Ql (106.54 meV). It is found that in dimer B, two *f*‐Pn‐6‐Ql molecules are stacked like the gears, which means one quinoline group of an *f*‐Pn‐6‐Ql molecule is inserted between two face‐to‐face stacked quinoline groups of another *f*‐Pn‐6‐Ql molecule. Such an arrangement can better take advantage of through‐space conjugation to strengthen intermolecular interactions, leading to effective ET. Dimer *α* of *l*‐Pn‐6‐Ql has a |*J*| value of 29.33 meV (Figure S6B, Supporting Information), which is comparable to *kT* value, indicating that the ET efficiency of this channel is greatly affected by thermal fluctuation. Therefore, dimer *α* of *l*‐Pn‐6‐Ql should not be as effective as dimers A and C of *f*‐Pn‐6‐Ql to serve as ET channel. So, it is obvious that *l*‐Pn‐6‐Ql has only one effective ET channel but *f*‐Pn‐6‐Ql has three effective ET channels along different directions, which strongly proves that *f*‐Pn‐6‐Ql has strong multi‐dimensional ET ability.

**Figure 4:**
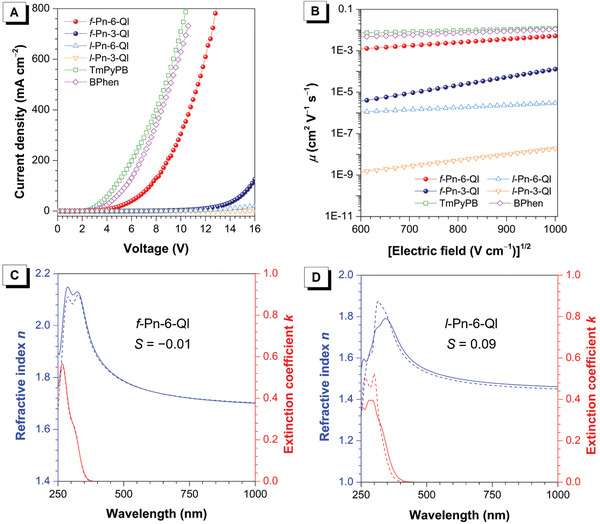
A) Plots of current density–voltage and B) *μ*_e_s of electron‐only devices for *f*‐Pn‐3‐Ql, *f*‐Pn‐6‐Ql, *l*‐Pn‐3‐Ql, *l*‐Pn‐6‐Ql, TmPyPB, and BPhen. Anisotropies in the refractive indices (blue) and the extinction coefficients (red) of the films of C) *f*‐Pn‐6‐Ql and D) *l*‐Pn‐6‐Ql. The solid and dash lines indicate horizontal and vertical components of optical constants, respectively.

Space charge limited current method^[^ ^18^ ^]^ is applied on electron‐only devices to evaluate the ET properties of folded molecules. Considering the energy levels of folded and linear molecules are close to the work function of Al surface modified by Cs_2_CO_3_ (2.1 eV),^[^ ^19^ ^]^ the configuration of electron‐only devices is designed as ITO/molecules (80 nm)/Cs_2_CO_3_ (2 nm)/Al. Herein, not only folded and linear molecules but also two widely used commercialized ET materials (TmPyPB and BPhen) are taken into comparison (**Figure** **4**A,B, Supporting Information). According to the current density‐voltage relationship (Figure 4A and Figure S1, Supporting Information), folded molecules show much larger current densities than the linear ones at the voltage range of 3–8 V. And the *μ*_e_s of folded molecules can be up to three orders of magnitude higher than those of the linear ones. For example, at the electrical field range from 6.4 × 10^5^ to 1.0 × 10^7^ V cm^−1^, the *μ*_e_s of *f*‐Pn‐6‐Ql are as high as 1.3 × 10^−3^ to 5.2 × 10^−3^ cm^2^ V^−1^ s^−1^, which are comparable to those of TmPyPB and BPhen, while the *μ*_e_s of *l*‐Pn‐6‐Ql are only 1.1 × 10^−6^ to 3.0 × 10^−6^ cm^2^ V^−1^ s^−1^ (Figure 4B). It is noticed that although *f*‐Pn‐6‐Ql and *f*‐Pn‐3‐Ql have similar folded molecular structures, the *μ*_e_ of *f*‐Pn‐6‐Ql is two orders magnitudes larger than that of *f*‐Pn‐3‐Ql. And the linear molecules also show the similar phenomena. According to previous reports,^[^ ^20^ ^]^ for the ET materials with heterocycle rings, like pyridine, quinoline, and phenanthroline, the coordination reaction between hetero atoms of ET materials and the electrode metals can influence the injection and mobility of electrons in ET layers. In comparison with *f*‐Pn‐3‐Ql (or *l*‐Pn‐3‐Ql), the *N* atoms of *f*‐Pn‐6‐Ql (or *l*‐Pn‐6‐Ql) are located at more outside positions, and the coordination reaction between *N* atoms and the electrode is easier because of less intramolecular steric hindrance. As a result, *f*‐Pn‐6‐Ql (or *l*‐Pn‐6‐Ql) has lower electron injection barrier and higher electron mobility than *f*‐Pn‐3‐Ql (or *l*‐Pn‐3‐Ql).

To further understand the ET mechanism of folded molecules, variable‐angle spectroscopic ellipsometry measurement is carried out to characterize the molecular alignment in film state (Figure 4C,D).^[^ ^21^ ^]^ When the transition dipole moments along the long axis of the molecule have a completely random orientation, orientation order parameter (*S*) estimated from the largest absorption peak is 0; when they have a completely horizontal orientation, *S* is −0.5; when they have completely vertical orientation, *S* is 1.^[^ ^22^ ^]^ The results show that *f*‐Pn‐6‐Ql has an *S* of −0.01, close to that of *l*‐Pn‐6‐Ql (0.09), suggesting that both molecules are deposited on the substrate with a random orientation. This means the excellent *μ*_e_ of *f*‐Pn‐6‐Ql is independent of molecular orientation. The worse through‐bond conjugation because of highly twisted configuration but significantly higher *μ*_e_ of *f*‐Pn‐6‐Ql further validate that the through‐space conjugation and efficient multi‐dimensional transport indeed exert positive impacts on ET.

**Figure 5:**
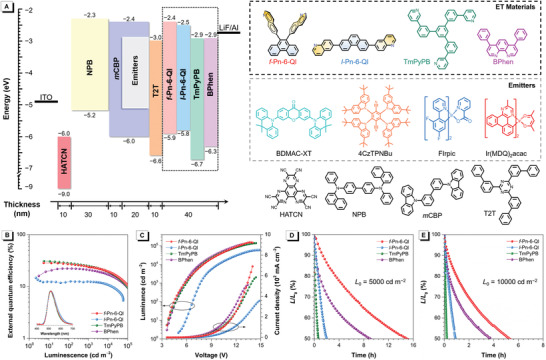
A) Device architecture, energy diagram, and functional layers for the vacuum‐deposited OLEDs. B) External quantum efficiency–luminance and C) luminance–voltage–current density characteristics of devices I–IV. Inset in plane B: EL spectra. Plots of relative luminance versus operation time measured at initial luminance of D) 5000 cd m^−2^ and E)10 000 cd m^−2^ for devices I–IV.

Considering the much better ET and easier preparation, *f*‐Pn‐6‐Ql is selected to investigate its performance as ET materials in OLEDs and the linear counterpart *l*‐Pn‐6‐Ql is adopted as a control. To comprehensively understand the ET behaviors of these molecules in OLEDs, two prevalent commercial ET materials, TmPyPB and BPhen, are also adopted as control. Four devices are fabricated with the configuration of ITO/2,3,6,7,10,11‐hexaazatriphenyl‐enehexacabonitrile (HATCN) (10 nm)/*N*,*N*′‐bis‐(1‐naphthalenyl)‐*N*,*N*′‐bis‐phenyl‐(1,1′‐biphenyl)‐4,4′‐diamine (NPB) (30 nm)/3,3′‐di(9*H*‐car‐bazol‐9‐yl)‐1,1′‐biphenyl (*m*CBP) (10 nm)/20 wt% 3,6‐bis(9,9‐dimethylacridin‐10‐yl)‐xanthen‐9‐one (BDMAC‐XT): *m*CBP (20 nm)/2,4,6‐tris(1,1′‐biphenyl)‐1,3,5‐triazine(T2T) (10 nm)/ET material (40 nm)/LiF (1 nm)/Al (**Figure** **5**A), in which ET layers are *f*‐Pn‐6‐Ql, *l*‐Pn‐6‐Ql, TmPyPB and BPhen for devices I–IV, respectively. In these devices, HATCN serves as hole‐injection layer, NPB serves as hole transport layer, and T2T serves as hole‐blocking layer. 3,6‐Bis(9,9‐dimethylacridin‐10(9H)‐yl)‐9H‐xanthen‐9‐one (BDMAC‐XT),^[^ ^23^ ^]^ a delayed fluorescence emitter is chosen as emitting layer and *m*CBP as host material in devices I–IV. These devices show similar EL spectra and close CIE coordinates (Figure 5B and **Table** **2**). Device I using *f*‐Pn‐6‐Ql as ET layer has a turn‐on voltage of 3.6 V, which is comparable with those of the devices using TmPyPB and BPhen, indicative of efficient electron injection and transport. This device shows an excellent maximum external quantum efficiency (*η*_ext,max_) of 28.4% and the largest maximum luminance (*L*_max_) of 154 600 cd m^−2^. The *LT*_50_ (time to 50% of initial luminance) values reach 15.3 and 5.2 h at high initial luminance of 5000 and 10 000 cd m^−2^, respectively. It is believed that, rigid structure of *f*‐Pn‐6‐Ql is beneficial to the thermal stability, increasing the stability of the devices as well. On the other hand, device II with *l*‐Pn‐6‐Ql as ET layer has a higher turn‐on voltage of 5 V, and only attains a lower *η*_ext,max_ of 14.6% and greatly decreased *LT*_50_ values of 0.9 and 0.7 h at initial luminance of 5000 and 10 000 cd m^−2^, respectively. The poor ET ability of *l*‐Pn‐6‐Ql leads to imbalanced carrier transport, which is accountable for the low efficiency and short device lifetime. In addition, the devices fabricated with commercial ET materials cannot realize high EL efficiency and long device lifetime at the same time neither (Table 2). Device III with TmPyPB as ET layer has a high *η*_ext,max_ of 30.5%, but the *LT*_50_ values are only 0.6 and 0.2 h at initial luminance of 5000 and 10 000 cd m^−2^, respectively. In comparison with device I, device IV with BPhen as ET layer provides inferior EL performances as well, with a lower *η*_ext,max_ of 22.2%, and shorter *LT*_50_ values of 8.9 and 3.7 h at initial luminance of 5000 and 10 000 cd m^−2^. The shorter device lifetimes of devices III and IV can be partially attributed to the low glass‐transition temperatures of TmPyPB and BPhen (<80 °C),^[^ ^3^, ^5^ ^]^ which lead to the morphological instability of the films.

**Table 2:** The key performance data of OLEDs

							Maximum value/at 1000 cd m^−2^			*L*_0_ = 5000/10 000 cd m^−2^
Device^a)^	EML	ETL	*V*_on_ [V]	*λ*_EL_ [nm]	CIE (x, y)	*L*_max_ [cd m^−2^]	*η*_C_ [cd A^−1^]	*η*_P_ [lm W^−1^]	*η*_ext_ [%]	*LT*_50_ [h]
I	BDMAC‐XT	*f*‐Pn‐6‐Ql	3.6	518	(0.261, 0.578)	154 600	89.8/75.2	78.3/43.7	28.4/23.8	15.3/5.2
II	BDMAC‐XT	*l*‐Pn‐6‐Ql	5.0	520	(0.280, 0.556)	59 160	45.5/38.2	28.6/15.4	14.6/12.3	0.9/0.7
III	BDMAC‐XT	TmPyPB	3.6	516	(0.250, 0.570)	139 500	95.4/76.9	78.8/40.2	30.5/24.9	0.6/0.2
IV	BDMAC‐XT	BPhen	3.6	516	(0.251, 0.569)	127 300	69.1/64.5	47.2/34.9	22.2/20.7	8.9/3.7
V	4CzTPNBu	*f*‐Pn‐6‐Ql	5.0	562	(0.477, 0.517)	78 080	84.0/62.0	52.7/23.4	25.8/19.0	8.0/2.3
VI	4CzTPNBu	TmPyPB	4.4	560	(0.456, 0.535)	55 430	60.1/44.9	42.0/18.1	18.2/13.8	0.3/0.1
VII	4CzTPNBu	BPhen	4.6	564	(0.470, 0.519)	75 070	46.9/41.6	24.5/17.7	14.5/13.2	11.1/3.8
VIII	FIrpic	*f*‐Pn‐6‐Ql	4.6	472	(0.157, 0.329)	53 510	26.4/26.4	16.5/10.1	13.1/13.0	0.6/0.2
IX	FIrpic	TmPyPB	4.0	470	(0.169, 0.325)	41 370	29.7/28.4	20.4/12.7	14.0/13.3	0.1/0.01
X	FIrpic	BPhen	4.2	472	(0.161, 0.332)	47 250	18.7/‐	7.5/‐	8.9/‐	0.3/0.1
XI	Ir(MDQ)_2_acac	*f*‐Pn‐6‐Ql	5.0	600	(0.598, 0.396)	88 450	38.6/28.4	30.3/9.8	21.0/13.4	4.7/1.3
XII	Ir(MDQ)_2_acac	TmPyPB	4.4	602	(0.580, 0.414)	30 940	31.6/20.9	22.6/8.0	14.8/9.8	0.1/0.04
XIII	Ir(MDQ)_2_acac	BPhen	4.6	604	(0.590, 0.404)	34 620	28.9/21.5	16.7/8.7	14.5/10.8	3.5/0.7

^a)^ Abbreviations: EML = emitting layer; ETL = electron transport layer; *V*_on_ = turn‐on voltage at 1 cd m^−2^; *λ*_EL_ = EL peak at 10 mA cm^−2^; CIE = Commission Internationale de l'Eclairage coordinates at 10 mA cm^−2^; *L*_max_ = maximum luminance; *η*_ext_/*η*_C_/*η*_P_ = external quantum efficiency/current efficiency/power efficiency; *LT*_50_ = the time required to decay 50% of initial luminance (*L*_0_).

To verify the universality of *f*‐Pn‐6‐Ql as ET material for OLEDs, it is further adopted as EL layer to fabricate OLEDs with other commercial luminescent materials such as blue delayed fluorescence emitter 2,3,5,6‐tetrakis(3,6‐di‐(*tert*‐butyl)carbazol‐9‐yl)‐1,4‐dicyanobenzene (4CzTPNBu) as well as blue and orange‐red phosphorescence emitters, bis[2‐(4,6‐difluoro‐phenyl)pyridinato‐*C* ^2^,*N*](picolinato)iridium(III) (FIrpic) and bis(2‐methyldibenzo‐[*f*,*h*]‐quinoxaline)Ir(III)(acetyl‐acetonate) (Ir(MDQ)_2_acac). For fair comparison, devices V–VII of 4CzTPNBu, devices VIII–X of FIrpic, and devices XI–XIII of Ir(MDQ)_2_acac are fabricated in the same configurations as those for BDMAC‐XT, and three kinds of ET materials of *f*‐Pn‐6‐Ql, TmPyPB, and BPhen are used for each emitter (Table 1 and Figure S9–S11, Supporting Information). The device V, VIII, and XI fabricated with *f*‐Pn‐6‐Ql as ET layer can achieve high EL efficiencies and long lifetimes simultaneously, with *η*_ext,max_s of 25.8, 13.1, and 21.0%, respectively, which are comparable to or even better than those of devices with TmPyPB and BPhen as ET layers. Besides, the *LT*_50_ values of device VIII and XI are the longest among the devices based on the same emitters of FIrpic and Ir(MDQ)_2_acac, respectively. These results strongly prove that *f*‐Pn‐6‐Ql is a promising candidate for fabricating OLEDs with high efficiency and long lifetime.

## 3 Conclusion

In conclusion, a proof‐of‐concept study is conducted for the exploration of robust ET materials to improve EL efficiencies and operational lifetimes of OLEDs. Novel thermally stable folded molecules with prominent through‐space conjugation (*f*‐Pn‐6‐Ql and *f*‐Pn‐3‐Ql) are created by bonding two quinolines to the 9,10‐positions of phenanthrene. They show higher *E*_T_s over 2.7 eV, and possess significantly improved ET ability with *μ*_e_s of two orders of magnitude higher than their linear counterparts (*l*‐Pn‐6‐Ql and *l*‐Pn‐3‐Ql). *f*‐Pn‐6‐Ql has a smaller *λ*_−_ than *l*‐Pn‐6‐Ql, owning to the higher molecular rigidity induced by enhanced intramolecular rotation barrier in the folded structure, and holds large |*J*| values between adjacent molecules at various directions, resulting in efficient multi‐dimensional ET, independent of molecular orientation. In addition, no matter using delayed fluorescence emitters or phosphorescence materials, the OLEDs with *f*‐Pn‐6‐Ql as ET layers achieve high EL efficiencies and long operational lifetimes at the same time, which are difficult to realize with *l*‐Pn‐6‐Ql or commercialized ET materials (TmPyPB and BPhen). These results reveal that the through‐space conjugated folded configuration could be a promising design of molecular framework for carrier transport materials to simultaneously achieve high EL efficiency and long operational lifetime for OLEDs. The merit of multi‐dimensional, orientation‐independent ET can also enable the ET materials designed by this strategy to find an array of applications in other electronic devices.

## Conflict of Interest

The authors declare no conflict of interest.

## Supporting information

Supporting Information

## Data Availability

The data that support the findings of this study are available from the corresponding author upon reasonable request.
