# Supplementary material for: Through‐Space Conjugated Electron Transport Materials for Improving Efficiency and Lifetime of Organic Light‐Emitting Diodes
Source: Adv Sci (Weinh). 2022 Mar 24;9(15):2200374. doi: 10.1002/advs.202200374 (PMC9130898; doi:10.1002/advs.202200374)
Supplement: Supplementary file 1 — Supporting Information [file ADVS-9-2200374-s001.pdf]

ADVANCED SCIENCE
Open Access

Supporting Information

for *Adv. Sci.*, DOI 10.1002/advs.202200374

Through-Space Conjugated Electron Transport Materials for Improving Efficiency and Lifetime of Organic Light-Emitting Diodes

Pingchuan Shen, Hao Liu, Zeyan Zhuang, Jiajie Zeng, Zujin Zhao and Ben Zhong Tang*

© 2022 Wiley-VCH GmbH

Supporting Information (SI)

General information

All other chemicals and reagents were purchased from commercial sources and used as received without further purification. ^{1}H and ^{13}C NMR spectra were measured on a Bruker AV 400 or 500 spectrometers in appropriated deuterated solution at room temperature. High-resolution mass spectra (HRMS) were recorded on a GCT premier CAB048 mass spectrometer operating in MALDI-TOF mode. High-performance liquid chromatography (HPLC) spectra were tested with Waters Alliance e2695 separations module, and methanol and acetonitrile used in the experiments were HPLC grade and they were purchased from Merck Ltd. Single crystals were tested on a Bruker D8 VENTURE Metaljet PHOTON III diffractometer and solved with Olex2 software.[1] To be specific, the crystal structure was solved with the SHELXT[2] structure solution program using Intrinsic Phasing and refined with the SHELXL[3] refinement package using Least Squares minimization. UV-vis absorption spectra were measured on a Shimadzu UV-2600 spectrophotometer. Photoluminescence (PL) spectra were recorded on a Horiba FluoroMax plus spectrofluorometer. Thermogravimetric analysis (TGA) was carried on a thermal gravimetric analyzer (Netzsch, TG209F1) under dry nitrogen at a heating rate of 20 ^{o}C min^{-1}. Differential scanning calorimetry (DSC) was run on a DSC (Netzsch, DSC 214 Polymer). DSC tests were carried out under dry nitrogen, and there were 6 temperature controlling process: a) 30 → 300 ^{o}C (20 ^{o}C min^{-1}); b) 300 ^{o}C (5 min); c) 300 → −30 ^{o}C (20 ^{o}C min^{-1}); d) −30 ^{o}C (5 min); e) −30 ^{o}C →300 ^{o}C (10 ^{o}C min^{-1}); f) 300 → 40 ^{o}C (40 ^{o}C min^{-1}) and curves from process e) were selected. Cyclic voltammograms were obtained in dichloromethane and *N*,*N*-dimethylformamide containing 0.1 M tetrabutylammonium hexafluorophosphate for oxidation and reduction processes, respectively, with a scan rate of 50 mV s^{-1}, using a platinum wire as the auxiliary electrode, a glass carbon disk as the working electrode and Ag/Ag^{+} as the reference electrode. $E_{HOMO} = [E_{ox} - E_{1/2}(Fc/Fc^{+}) + 4.8]$ eV, $E_{LUMO} = [E_{red} - E_{1/2}(Fc/Fc^{+}) + 4.8]$ eV, where E_{ox} and E_{red} represent the onset oxidation potential and the reduction potential relative to Fc/Fc^{+} (4.8 eV), respectively, and $E_{1/2}(Fc/Fc^{+})$ represents the calibrated value.

Synthesis and characterization

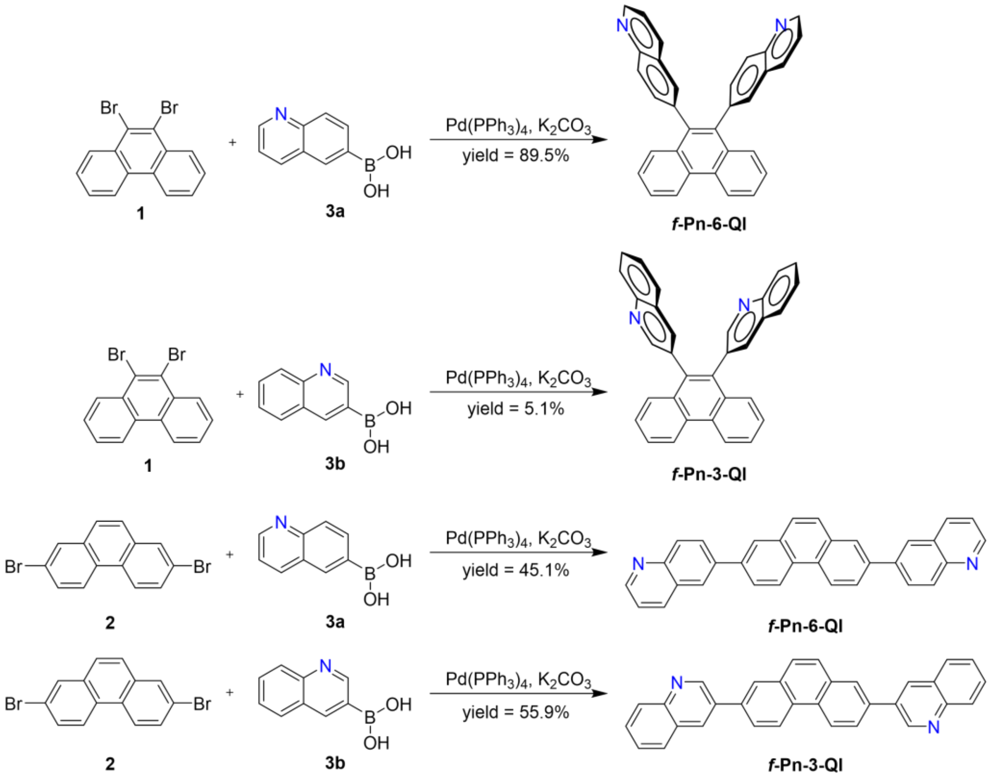

Scheme S1. Synthetic routes of *f*-Pn-6-Ql, *f*-Pn-3-Ql, *l*-Pn-6-Ql and *l*-Pn-3-Ql.

Synthesis procedures of folded molecules: Into a 500 mL two-necked round bottom flask was placed **1** (5.0 g, 14.9 mmol), **3a**/**3b** (10.3 g, 59.6 mmol), $Pd(PPh_3)_4$ (1.7 g, 1.5 mmol) and K_2CO_3 (16.5 g, 119.2 mmol). The flask was evacuated under vacuum and flushed with dry nitrogen three times, and then a mixture of methylbenzene, ethyl alcohol and water (150 mL, 2/1/1, *v*/*v*/*v*) was added. The reaction mixture was refluxed for 6 h. After cooling to room temperature, the mixture was poured into water and extracted with dichloromethane three times. After solvent evaporation, the crude product was purified by silica-gel column chromatography with dichloromethane and ethyl acetate as eluent. White solids of *f*-Pn-6-Ql and *f*-Pn-3-Ql were isolated in 89.8% and 5.1% yields, respectively. The obtained products were subject to further purification by temperature-gradient sublimation before property measurement and OLED fabrication.

***f*-Pn-6-Ql**: ^{1}H NMR (400 MHz, DMSO-d_6) δ 9.06 (d, J = 8.0 Hz, 2H), 8.84−8.78 (m, 2H), 8.26−8.17 (m, 2H), 7.91−7.82 (m, 4H), 7.81−7.74 (m, 2H), 7.70−7.65 (m, 2H), 7.61−7.53 (m, 2H), 7.49−7.35 (m, 4H). ^{13}C NMR (125 MHz, $CDCl_3$) δ 171.28, 150.50, 150.45, 147.08, 146.97, 138.06, 137.96, 136.95, 136.84, 136.29, 136.03, 132.96, 132.88, 131.76, 130.34, 130.31, 129.89, 129.63, 129.05, 128.74, 127.95, 127.84, 127.09, 127.01, 122.84, 121.49, 121.42. HRMS ($C_{32}H_{20}N_2$): *m/z* 433.1731 (M + H$^+$, calcd 433.1705).

***f*-Pn-3-Ql**: ^{1}H NMR (500 MHz, DMSO-d_6) δ 9.10 (d, J = 8.5 Hz, 2H), 8.84 (d, J = 2.0 Hz, 1H), 8.74 (d, J = 2.0 Hz, 1H), 8.43 (d, J = 1.5 Hz, 1H), 8.31 (d, J = 1.5 Hz, 1H), 7.96−7.87 (m,

3H), 7.87−7.77 (m, 3H), 7.74−7.67 (m, 2H), 7.65−7.56 (m, 3H), 7.45−7.36 (m, 1H), 7.44−7.37 (m, 2H). ^{13}C NMR (125 MHz, $CDCl_3$) δ 152.23, 152.19, 146.97, 146.82, 137.83, 137.74, 134.89, 134.84, 132.29, 132.28, 131.71, 130.56, 130.55, 129.88, 129.74, 129.54, 129.22, 128.09, 127.75, 127.60, 127.56, 127.49, 127.48, 127.40, 127.36, 127.20, 127.09, 123.02. HRMS ($C_{32}H_{20}N_2$): *m/z* 433.1699 (M + H^+, calcd 433.1705).

Crystal data for *f*-Pn-6-Ql (CCDC 2056395): Single crystals of *f*-Pn-6-Ql were grown in dichloromethane by slow solvent evaporation. $C_{32}H_{20}N_2$, M_W = 432.50, P −1, a = 9.554(10), b = 10.3954(11), c = 111.8284(14) Å, V = 1104.7(2) Å^3, Z = 2, D_c = 1.300 g cm^{-3}, μ = 0.080 mm^{-1} (MoKα, λ = 1.34139), F(000) = 1088, T = 173.00 K, $2\theta_{max}$ = 60.218^o (95.2%), 13276 measured reflections, 4785 independent reflections (R_{int} = 0.0833), GOF on F^2 = 1.059, R_1 = 0.1618, wR_2 = 0.2928 (all data), Δe 0.440 and −0.406 eÅ^{-3}.

Synthesis procedures of linear molecules: Into a 250 mL two-necked round bottom flask was placed **2** (1.0 g, 3.0 mmol), **3a**/**3b** (2.1 g, 12 mmol), $Pd(PPh_3)_4$ (0.35 g, 0.3 mmol) and K_2CO_3 (2.5 g, 18 mmol). The flask was evacuated under vacuum and flushed with dry nitrogen three times and then a mixture of methylbenzene, ethyl alcohol and water (80 mL, 2/1/1, v/v/v) was added. The reaction mixture was refluxed for 6 h. After cooling to room temperature, crude products were obtained by filtration and washing with tetrahydrofuran (100 mL). Temperature-gradient sublimation was carried out for further purification, and yellow green solids of *l*-Pn-6-Ql/*l*-Pn-3-Ql were isolated in 45.1%/55.9% yields.

***l*-Pn-6-Ql**: ^{1}H NMR (400 MHz, $CDCl_3$) δ 8.97 (d, J = 4.4 Hz, 2H), 8.86 (d, J = 8.4 Hz, 2H), 8.35−8.14 (m, 10H), 8.09 (d, J = 8.6 Hz, 2H), 7.92 (s, 2H), 7.51−7.46 (m, 2H). The ^{13}C NMR data are not available because of the poor solubility. HRMS ($C_{32}H_{20}N_2$): *m/z* 433.1717 (M + H^+, calcd 433.1705).

***l*-Pn-3-Ql**: ^{1}H NMR (400 MHz, CD_2Cl_2) δ 9.39 (d, J = 2.4 Hz, 2H), 8.91 (d, J = 8.8 Hz, 2H), 8.55 (d, J = 1.6 Hz, 2H), 8.34 (d, J = 1.6 Hz, 2H), 8.21−8.10 (m, 4H), 8.03−7.93 (m, 4H), 7.81−7.73 (m, 2H), 7.68−7.60 (m, 2H). The ^{13}C NMR data are not available because of the poor solubility. HRMS ($C_{32}H_{20}N_2$): *m/z* 433.1701 (M + H^+, calcd 433.1705).

Crystal data for *l*-Pn-6-Ql (CCDC: 2154379): Single crystals of *l*-Pn-6-Ql were grown in dichloromethane by slow solvent evaporation. $C_{32}H_{20}N_2$, M_W = 432.50, P b c a, a = 7.3055(2), b = 7.0472(2), c = 41.3949(11) Å, V = 2131.15(10) Å^3, Z = 4, D_c = 1.348 g cm^{-3}, μ = 0.607 mm^{-1} (CuKα, λ = 1.54178), F(000) = 904, T = 212.00 K, $2\theta_{max}$ = 68.266^o (99.7%), 25647 measured reflections, 1950 independent reflections (R_{int} = 0.0678), GOF on F^2 = 1.086, R_1 = 0.0450, wR_2 = 0.1104 (all data), Δe 0.214 and −0.252 eÅ^{-3}.

Computational details

Theoretical calculations were carried out by Gaussian 16 (B.01) program.[4] Geometry optimization and relax scan were implemented using B3LYP exchange-correlation functional with Grimme's DFT-D3(BJ) empirical dispersion correction, which abbreviated as B3LYP-D3(BJ) in conjunction with def2-TZVP basis set. Electrostatic potentials (ESP) on the basis of the optimized geometry and wavefunction at B3LYP-D3(BJ)/def2-TZVP level were performed with the Multiwfn 3.8 code developed by Lu's group. In order to obtain accurate reorganization energy (λ_{-}), M06-2X functionals and ma-QZVP basis, which is the "minimally augmented" version of the def2-QZVP basis set were employed in single point calculations.

To calculate the charge transfer integral *J* values of dimers in crystals, it is necessary to do the geometry optimization to correct the C−H bond length in crystals, because the position of H atoms obtained from XRD are inaccurate. Geometry optimizations of 7 different dimers were carried out at B3LYP-D3(BJ)/def2TZVP level with C and N atoms frozen. Then, the single point calculation of dimers and two monomers in each dimer were carried out based on the optimized geometries. Dispersion function played an essential role in the calculation of *J*, however, convergence criterions were unable to be met in self-consistent field iteration when doing single point calculation of the dimers using large basis set such as ma-TZVP(-f), ma-TZVP and ma-QZVP, probably due to the dimers' geometries in crystal were different from those in gas phase. Hence, the single point calculation of the dimers was carried out at M06-2X/ma-SVP level. Last, program coded for *J* calculation based on the methods provided in the ref[16] of manuscript are downloaded from http://bbs.keinsci.com/thread-11369-1-1.html to calculate the *J* value with the single point calculation results of the monomers and dimers.

Isosurface maps of ESP and molecular orbitals were rendered by means of Visual Molecular Dynamics (VMD) software based on the files exported by Multiwfn. Since solvent effect was not the focus of this work, all studies were implemented in vacuum.

Carrier mobility measurement

The space charge limited current (SCLC) property can be described via the Mott-Gurney equation (**Equation. S1**), and the electron mobility (μ_e) of organic semiconductor can be calculated according to the Poole-Frenkel formula (**Equation. S2**), where ε_0 is the free-space permittivity (8.85×10^{-14} C V^{-1} cm^{-1}), ε_r is the relative dielectric constant (assumed to be 3.0 for organic semiconductor), *E* is the electric field, μ_0 is the zero-field mobility, γ is the Poole-Frenkel factor, and *L* is the thickness of the neat film of each molecule. As shown in **Figure S1**, the curves exhibit prominent ohmic characteristics, while upon the increase of voltage, the currents become space-charge limited. By fitting the current density–voltage curves in the

SCLC region, according to Equation. S1, the μ_0 and γ values are obtained, thus generating the field-dependent carrier mobility by Equation. S2.

$$J = \frac{9}{8}\varepsilon_0\varepsilon_r\mu\frac{E^2}{L} = \frac{9}{8}\varepsilon_0\varepsilon_r\frac{V^2}{L^3}\mu_0 e^{\left(0.891\gamma\sqrt{\frac{V}{L}}\right)} \text{ (Equation. S1)}$$

$$\mu_e = \mu_0 e^{(\gamma\sqrt{E})} \text{ (Equation. S2)}$$

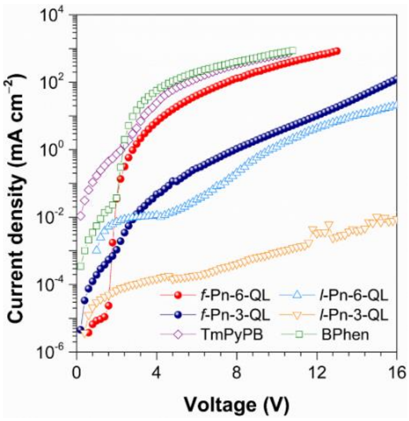

Figure S1. Log-scale plot of current density–voltage of electron-only devices for *f*-Pn-6-Ql, *f*-Pn-3-Ql, *l*-Pn-6-Ql, *l*-Pn-3-Ql, TmPyPB and BPhen.

OLED fabrication and measurement

The glass substrates precoated with a 90-nm layer of indium tin oxide (ITO) with a sheet resistance of 15 to 20 ohms per square were successively cleaned in ultrasonic bath of acetone, isopropanol, detergent, and deionized water, respectively, taking 10 min for each step. Then, the substrates were completely dried in a 70 °C oven. Before the fabrication processes, to improve the hole injection ability of ITO, the substrates were treated by O_2 plasma for 10 min. The vacuum-deposited OLEDs were fabricated under a pressure of $<5 \times 10^{-4}$ Pa in a Fangsheng OMV-FS450 vacuum deposition system. Organic materials, LiF, and Al were deposited at rates of 1 to 2 Å s^{-1}, 0.1 Å s^{-1}, and 5 Å s^{-1}, respectively. The effective emitting area of the device was 9 mm^2, determined by the overlap between anode and cathode. The luminance–voltage–current density and external quantum efficiency were characterized with a dual-channel Keithley 2614B source meter and a PIN-25D silicon photodiode. The EL spectra were obtained via an Ocean Optics USB 2000+ spectrometer, along with a Keithley 2614B source meter. All the characterizations were conducted at room temperature in ambient conditions without any encapsulation, as soon as the devices were fabricated. The OLED device lifetimes measurement were carried out using PIN-25D silicon photodiode under nitrogen atmosphere in the glovebox, without special treatment such as packaging.

Ellipsometry Measurements: Variable angle spectroscopic ellipsometry (VASE) measurements for the films on Si substrates were performed using a fast spectroscopic

ellipsometer (M-2000U, J. A. Woollam Co., Inc.) at seven angles of incident light from 45° to 75° in steps of 5°. At each angle, the experimental ellipsometric parameters Ψ and Δ were simultaneously obtained in steps of 1.6 nm throughout the spectral region from 250 nm to 1000 nm. The analysis of all the combined VASE data was performed using the "WVASE32" software (J. A. Woollam Co., Inc). The method used to determine the anisotropic optical constants *S* is described in detail in reference.[5]

Additional data

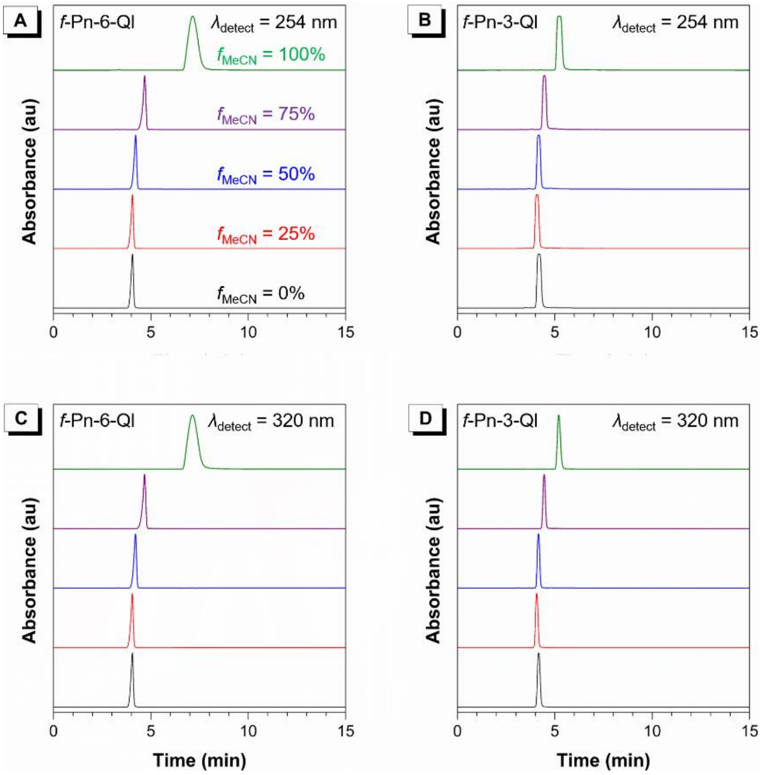

Figure S2. HPLC spectra monitored at 254 and 320 nm with acetonitrile-methanol ratio varying from 0/100 to 100/0 (v/v) for (A and C) *f*-Pn-6-Ql and (B and D) *f*-Pn-3-Ql.

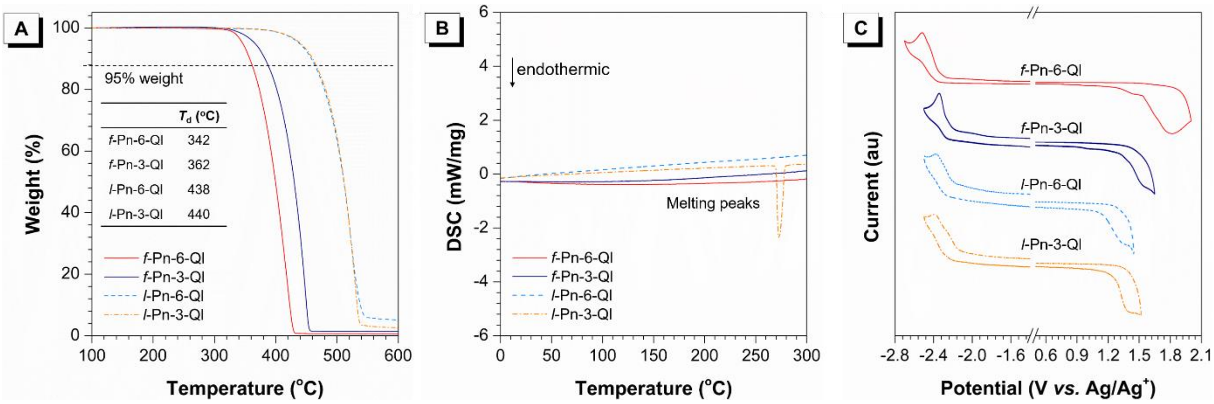

Figure S3. (A) Thermogravimetric analysis, and (B) differential scanning calorimetry curves and (C) cyclic voltammograms of *f*-Pn-6-Ql, *f*-Pn-3-Ql, *l*-Pn-6-Ql and *l*-Pn-3-Ql. The

decomposition temperatures (T_ds) of *f*-Pn-6-Ql, *f*-Pn-3-Ql, *l*-Pn-6-Ql and *l*-Pn-3-Ql are indicated in plane A.

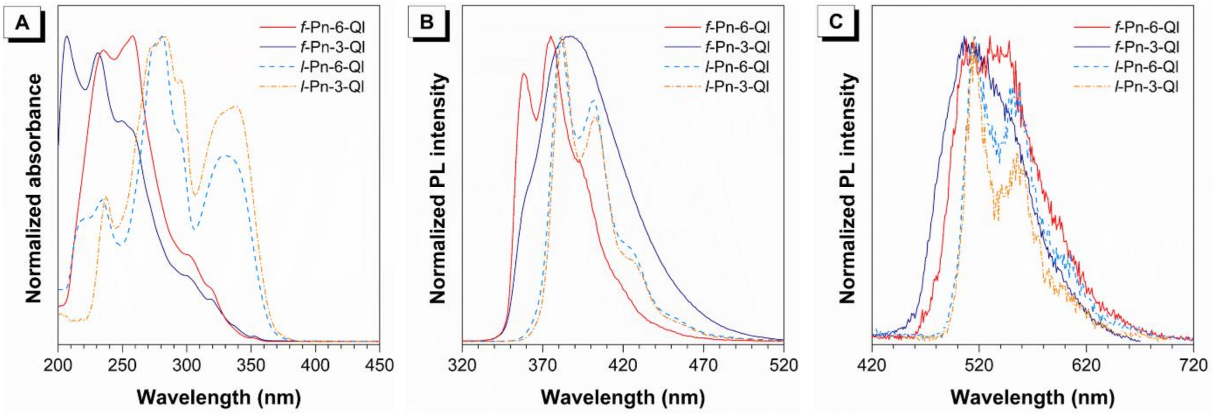

Figure S4. Normalized (A) UV-vis absorption, (B) fluorescence, and (C) phosphorescence spectra of *f*-Pn-6-Ql, *f*-Pn-3-Ql, *l*-Pn-6-Ql and *l*-Pn-3-Ql tested in dilute THF solution with a concentration of 10^{-5} M.

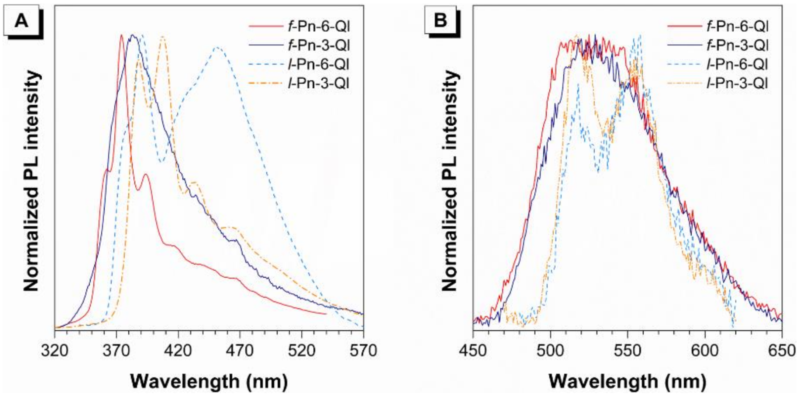

Figure S5. Normalized (A) fluorescence and (B) phosphorescence spectra of *f*-Pn-6-Ql, *f*-Pn-3-Ql, *l*-Pn-6-Ql and *l*-Pn-3-Ql in neat films.

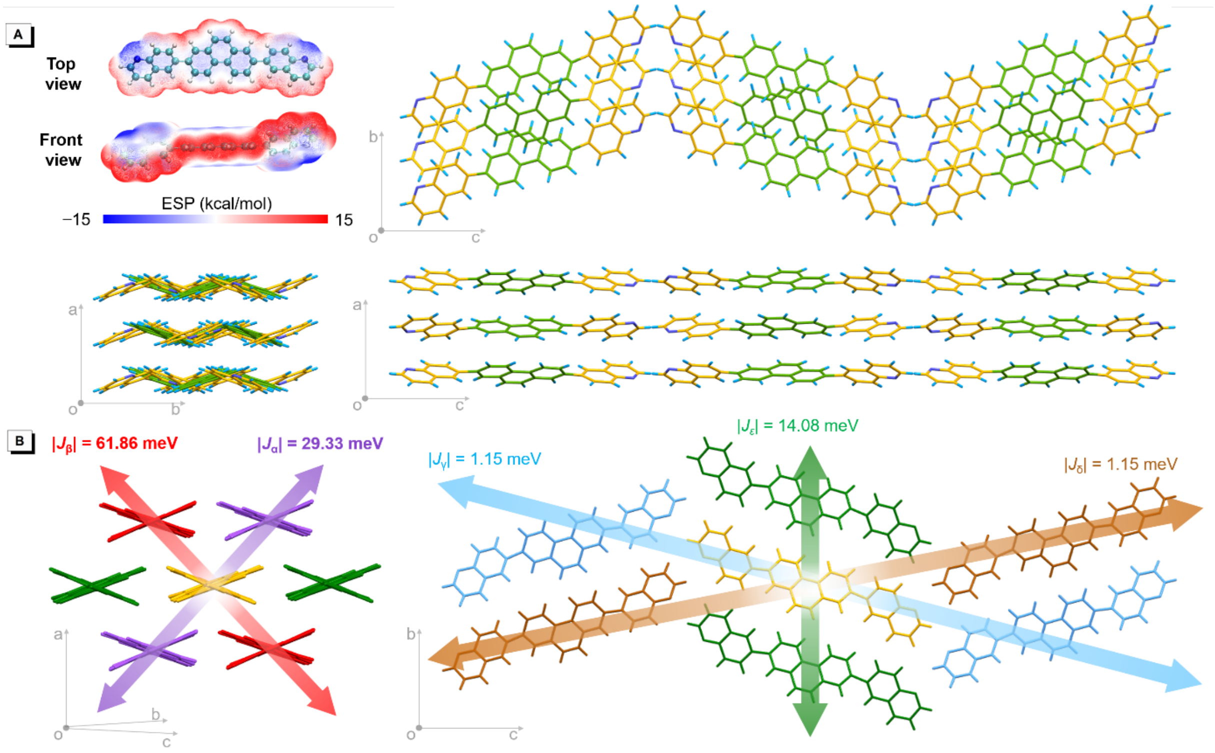

Figure S6. (A) Isosurface of ESP and packing arrangements of *l*-Pn-6-Ql in crystal. (B) Calculated $|J|$ values for *l*-Pn-6-Ql. $|J|$ values at different directions are labeled in different colors.

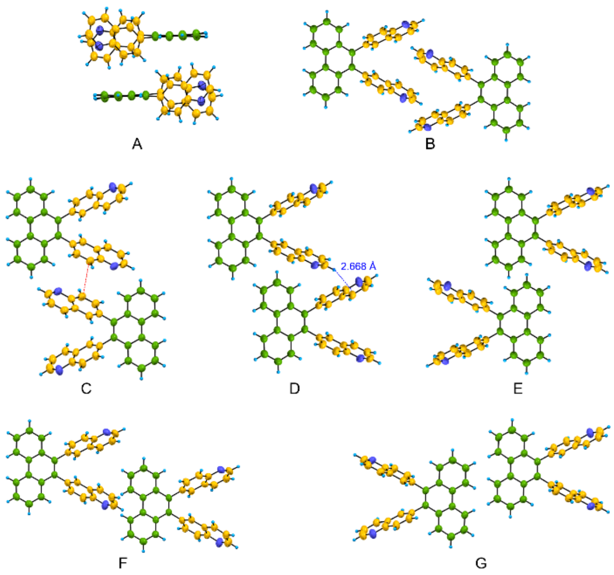

Figure S7. Seven different dimers in the crystal of *f*-Pn-6-Ql according to the relative intermolecular positions between central molecules and the adjacent ten molecules.

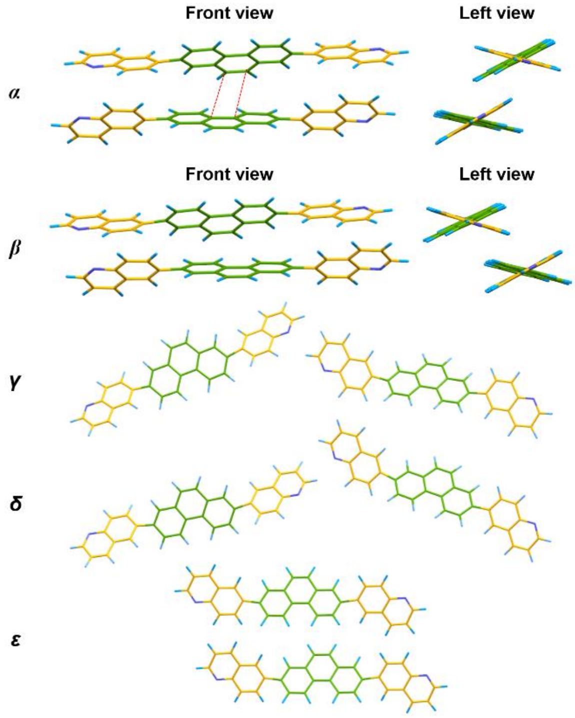

Figure S8. Five different dimers in the crystal of *l*-Pn-6-Ql according to the relative intermolecular positions between central molecules and the adjacent ten molecules.

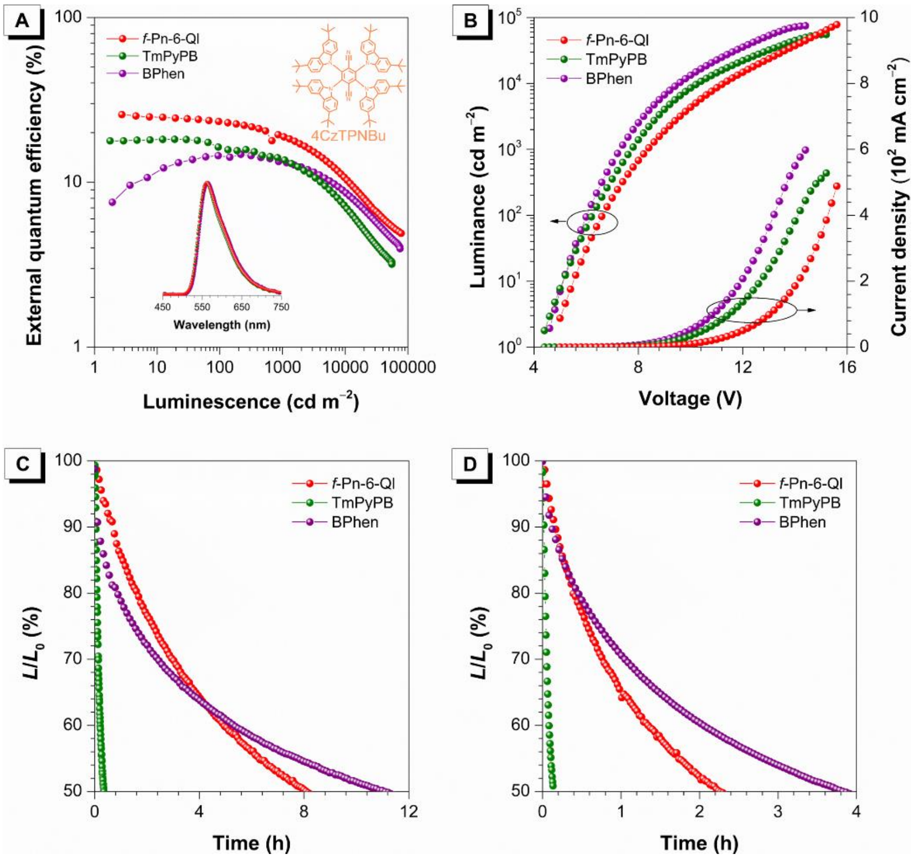

Figure S9. Device performance of OLED V−VII with device configurations of: ITO/HATCN (10 nm)/NPB (30 nm)/*m*CBP (10 nm)/5 wt% 4CzTPNBu: *m*CBP (20 nm)/T2T (10 nm)/ETL

(40 nm)/LiF (1 nm)/Al. ETL: electron transport layer. (A) External quantum efficiency–luminance and (B) luminance–voltage–current density characteristics. Inset in (A): EL spectra of the devices. Plots of relative luminance versus operation time measured at initial luminance of (C) 5000 cd m^{-2} and (D)10000 cd m^{-2}.

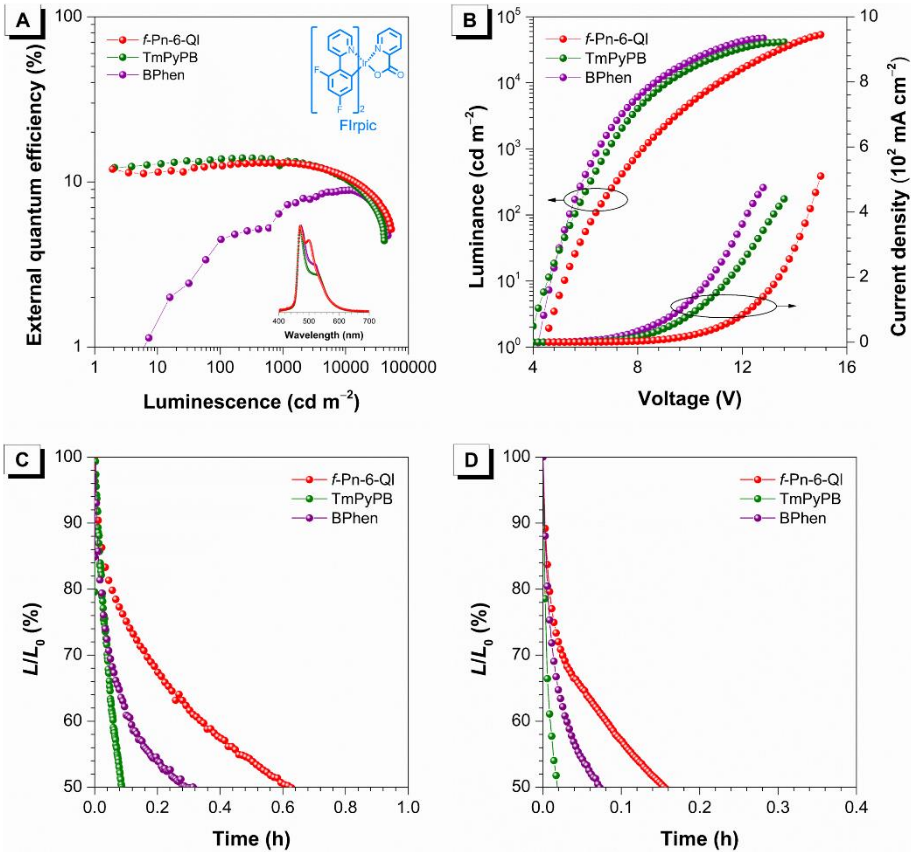

Figure S10. Device performance of OLED VIII−X with device configurations of: ITO/HATCN (10 nm)/NPB (30 nm)/*m*CBP (10 nm)/10 wt% FIrpic: *m*CBP (20 nm)/T2T (10 nm)/ETL (40 nm)/LiF (1 nm)/Al. ETL: electron transport layer. (A) External quantum efficiency–luminance and (B) luminance–voltage–current density characteristics. Inset in (A): EL spectra of the devices. Plots of relative luminance versus operation time measured at initial luminance of (C) 5000 cd m^{-2} and (D)10000 cd m^{-2}.

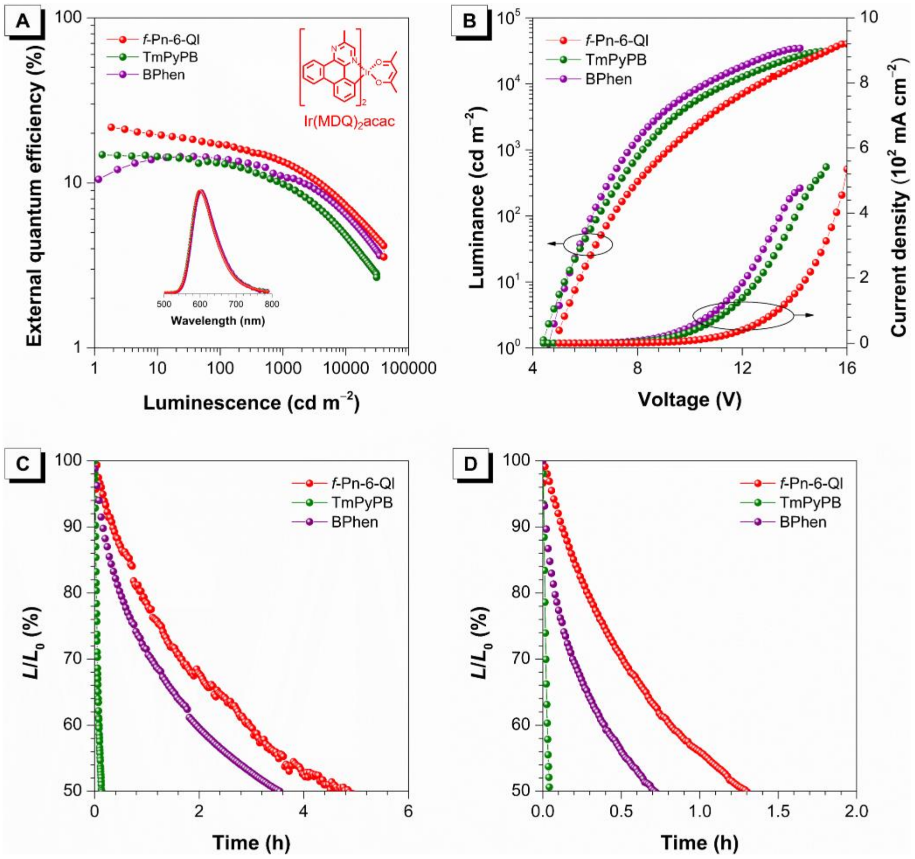

Figure S11. Device performance of OLED XI−XIII with device configurations of: ITO/HATCN (10 nm)/NPB (30 nm)/*m*CBP (10 nm)/3 wt% Ir(MDQ)$_2$acac: *m*CBP (20 nm)/T2T (10 nm)/ETL (40 nm)/LiF (1 nm)/Al. ETL: electron transport layer. (A) External quantum efficiency–luminance and (B) luminance–voltage–current density characteristics. Inset in (A): EL spectra of the devices. Plots of relative luminance versus operation time measured at initial luminance of (C) 5000 cd m^{-2} and (D)10000 cd m^{-2}.

References

[1] O. V. Dolomanov, L. J. Bourhis, R. J. Gildea, J. A. K. Howard, H. J. Puschmann, *Appl. Cryst.* **2009**, 42, 339–341.

[2] G. M. Sheldrick, *Acta Cryst.* **2015**, A71, 3–8.

[3] G. M. Sheldrick, *Acta Cryst.* **2015**, C71, 3–8.

[4] M. J. Frisch, G. W. Trucks, H. B. Schlegel, G. E. Scuseria, M. A. Robb, J. R. Cheeseman, G. Scalmani, V. Barone, G. A. Petersson, H. Nakatsuji, X. Li, M. Caricato, A. V. Marenich, J. Bloino, B. G. Janesko, R. Gomperts, B. Mennucci, H. P. Hratchian, J. V. Ortiz, A. F. Izmaylov, J. L. Sonnenberg, D. Williams-Young, F. Ding, F. Lipparini, F. Egidi, J. Goings, B. Peng, A. Petrone, T. Henderson, D. Ranasinghe, V. G. Zakrzewski, J. Gao, N. Rega, G. Zheng, W. Liang, M. Hada, M. Ehara, K. Toyota, R. Fukuda, J. Hasegawa, M. Ishida, T.

Nakajima, Y. Honda, O. Kitao, H. Nakai, T. Vreven, K. Throssell, J. A. Montgomery, Jr., J. E. Peralta, F. Ogliaro, M. J. Bearpark, J. J. Heyd, E. N. Brothers, K. N. Kudin, V. N. Staroverov, T. A. Keith, R. Kobayashi, J. Normand, K. Raghavachari, A. P. Rendell, J. C. Burant, S. S. Iyengar, J. Tomasi, M. Cossi, J. M. Millam, M. Klene, C. Adamo, R. Cammi, J. W. Ochterski, R. L. Martin, K. Morokuma, O. Farkas, J. B. Foresman, and D. J. Fox, *Gaussian, Inc., Wallingford CT*, **2016**.

[5] a) D. Yokoyama, A. Sakaguchi, M. Suzuki, C. Adachi, *Org. Electon.* **2009**, *10*, 127–137; b) J. Frischeisen, D. Yokoyama, C. Adachi, W. Brütting, *Appl. Phys. Lett.* **2010**, *96*, 073302.

Author Contributions

The manuscript was written through contributions of all authors. / All authors have given approval to the final version of the manuscript.
